# Pure and Confounded Effects of Causal SNPs on Longevity: Insights for Proper Interpretation of Research Findings in GWAS of Populations with Different Genetic Structures

**DOI:** 10.3389/fgene.2016.00188

**Published:** 2016-11-08

**Authors:** Anatoliy I. Yashin, Ilya Zhbannikov, Liubov Arbeeva, Konstantin G. Arbeev, Deqing Wu, Igor Akushevich, Arseniy Yashkin, Mikhail Kovtun, Alexander M. Kulminski, Eric Stallard, Irina Kulminskaya, Svetlana Ukraintseva

**Affiliations:** Biodemography of Aging Research Unit, Social Science Research Institute, Duke UniversityDurham, NC, USA

**Keywords:** linkage disequilibrium, population stratification, mortality selection, lack of replication, causal SNP, longevity related traits, population genetic structure

## Abstract

This paper shows that the effects of causal SNPs on lifespan, estimated through GWAS, may be confounded and the genetic structure of the study population may be responsible for this effect. Simulation experiments show that levels of linkage disequilibrium (LD) and other parameters of the population structure describing connections between two causal SNPs may substantially influence separate estimates of the effect of the causal SNPs on lifespan. This study suggests that differences in LD levels between two causal SNP loci within two study populations may contribute to the failure to replicate previous GWAS findings. The results of this paper also show that successful replication of the results of genetic association studies does not necessarily guarantee proper interpretation of the effect of a causal SNP on lifespan.

## Introduction

The results of many genome-wide association studies (GWAS) of complex traits suffer from a lack of replication (Shen et al., 2005; Gorroochurn et al., 2007; Greene et al., 2009; Hart et al., 2013; Ioannidis, 2015; Maxwell et al., 2015; Torrico et al., 2016). Differences in population genetic structures among study populations are considered to be possible contributors to this problem (Greene et al., 2009). One aspect of population structure—the differences in genetic frequencies among subgroups of individuals comprising the population—was traditionally linked with the effects of population stratification (Wacholder et al., 2000; Price et al., 2006, 2010). Another one—the presence of linkage disequilibrium (LD) in many parts of the human genome including those that contain causal SNPs—was actively exploited in GWAS of complex traits (Cantor et al., 2010; Moore et al., 2010; Hayes, 2013). Methods of fine mapping following the “discovery” phase are used for evaluating causal SNPs (Clarke et al., 2007; Hassanein et al., 2010; Zapata, 2013; Kichaev et al., 2014; Morris, 2014). One could expect that the non-replication problem due to differences in LD patterns among study populations in GWAS would disappear if the detected marker SNP is a causal one, i.e., if it contributes to the variability of a trait. It turns out that the differences in LD levels around a functional SNP may still contribute to the non-replication problem. The estimated associations in this case depend on whether the detected functional SNP is in LD with another functional SNP, the effects of these SNPs on the trait in the absence of LD (pure effects), and on the level of LD between corresponding SNP loci. This property has important consequences for interpretation of the results of genetic analyses of complex traits. In the presence of LD the estimated effects of a causal SNP may be spurious and may incorrectly characterize the biological relationships between the SNP and the trait. In contrast the pure effect of a given causal SNP estimated in the absence of LD with other such SNPs may correctly characterize the biological connections between the SNP and the trait. Therefore, for example, performing genetic analyses of African populations (that have lower levels of LD patterns for many SNP pairs than populations of European origin) has the potential to reduce bias in the estimated effects of functional SNPs on a trait caused by the presence of LD between functional loci (Shifman et al., 2003). This condition is, however, not sufficient because of the possible presence of hidden gene/gene interaction effects, gene/environment correlations, and gene/environment interaction effects (Ukraintseva et al., 2016).

Human lifespan and many other aging, health and longevity related traits are multifactorial phenotypes, that is, they are affected by many genetic and non-genetic factors. The relationships between genes and these phenotypes have special features that distinguish them from other complex traits, influence methods of their genetic analyses, and affect the interpretation of the research results. The genetic variants that influence aging, health, and longevity related traits generate age dependent changes in the population genetic structure, i.e., changes in the frequencies of genetic variants and in the levels of linkage disequilibrium (LD) among them. This feature has important implications for studies focused on the replication of GWAS research findings: Independent populations involved in such studies often have different genetic structures, due in part to the differences in the population age distribution at the time of biospecimen collection. As a result, the frequencies of the genetic variants associated with these traits and their LD patterns may differ even if the genetic structures in the corresponding population cohorts were the same at birth.

Detecting statistically significant associations of genetic variants with complex traits is not the end of the genetic analyses. One reason is that the relationship between a detected marker SNP and the complex trait of interest is not, necessarily, a causal one. More often these relationships serve as proxies for the real effect of some unobserved causal SNPs [due to linkage disequilibrium (LD) between the marker and causal SNPs], and, hence, do not have a direct biological effect on the phenotype. To generate insights about the biological mechanisms responsible for the trait's variability one has to identify the causal SNPs responsible for the association signal. To identify such SNPs a number of efficient fine-mapping procedures have been recommended (Zaitlen et al., 2010; Hormozdiari et al., 2015). The main limitation of existing methods is that they seek to identify a single causal variant which is independent of (not in LD with) other causal variants (Hormozdiari et al., 2014). Since this is not sufficiently realistic, a new approach that allows for efficient detection of multiple causal variants has been proposed (Hormozdiari et al., 2014). The case where two or more causal SNPs are in LD creates additional problems for interpretation of the results of genetic association studies.

In this paper we show that the estimates of the effects of a causal SNP on lifespan depend on the genetic structure of the population under study (e.g., the level of LD of the SNP with other causal SNPs). Genetic association studies of this trait using data from populations with different LD levels are likely to produce different results. We show that differences in population genetic structures can explain why genetic variants favorable for longevity in one population appear as harmful risk factors in another population. Population structure may also be responsible for the age-specific effects of genetic variants on mortality risk. Differences in genetic structures in distinct populations may be responsible for the low level of replicability of GWAS of human aging, health, and longevity related traits.

## Data and methods

**Figure 1:**
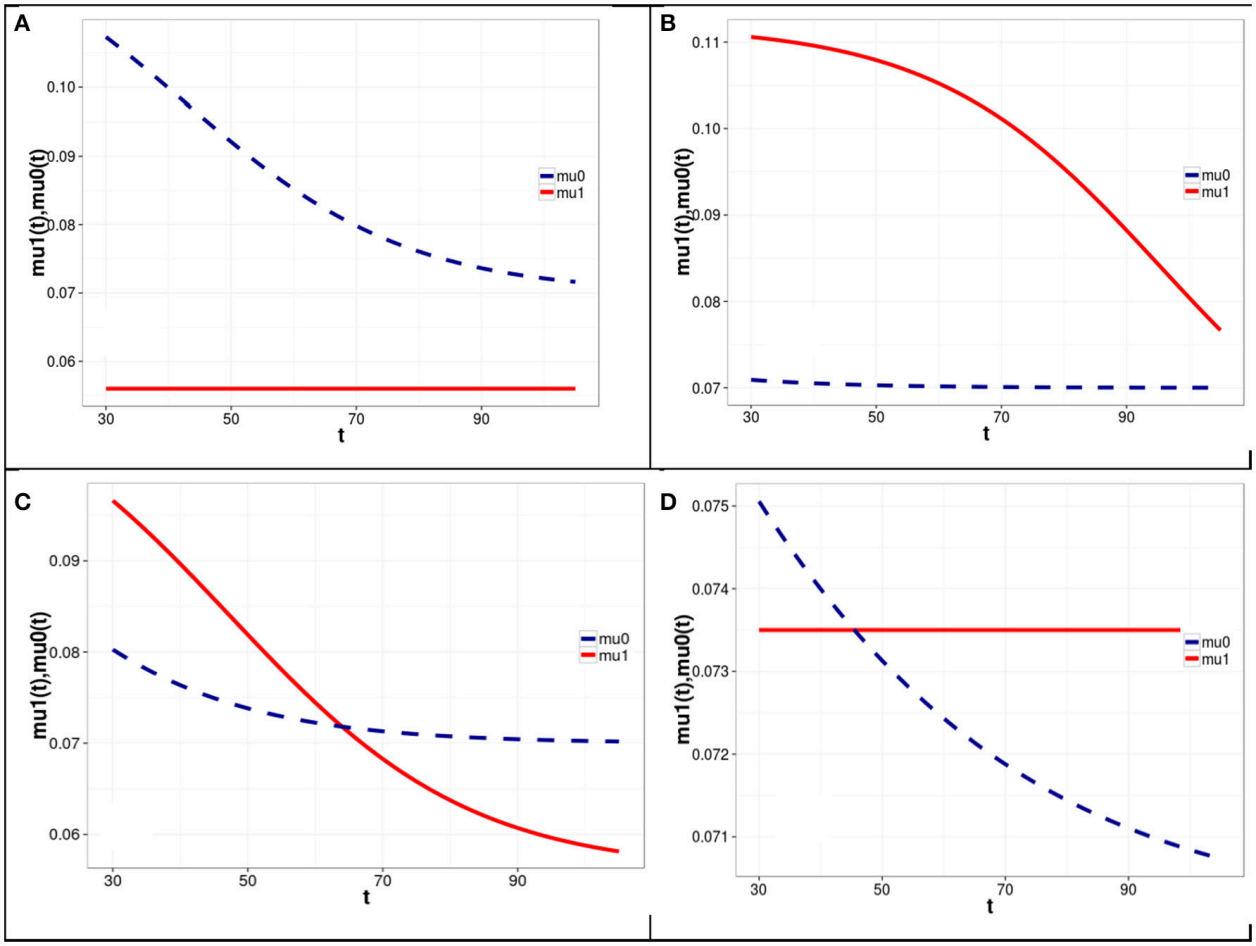
**Graphs of mortality rates for carriers (solid line) and non-carriers (dashed line) of the minor allele at the first SNP locus corresponding to different levels of LD between the two loci and different haplotype frequencies in four simulation experiments with Model 1 of the genetic influence on mortality rates**. Model parameters corresponding to the graphs shown in **(A–D)** are represented in Table 1 in rows **(A–D)**, respectively. Equations linking the mortality rates for the carriers and non-carriers of the minor allele of the first SNP (shown in **A–D** of Figure 1) with the mortality risks for haplotypes are given on last page of the Supplementary Materials.

To show how the effects of differences in LD levels and other parameters of the population genetic structure influence the results of genetic association studies we consider lifespan as the trait of interest and two causal SNP loci with minor and major alleles at each locus. Accordingly we will use a simple model of the genetic connections with lifespan that requires only dichotomous genetic variables (e.g., this is the case when the minor alleles at each locus have dominant genetic effects on lifespan). Extensions for cases with SNP genotypes are straightforward. Let *V*_1_ = (0, 1) and *V*_2_ = (0, 1) be the values of the genetic variants at these loci, where “1” denotes the minor allele and “0” corresponds to the major allele (see Figure 1S, Supplementary Materials). The genetic variants affect survival through mortality risks specified for haplotypes (*i,j*) where *i* = 0, 1 characterizes the presence of major or minor alleles in the first SNP locus, and *j* = 0, 1 describes the presence of such alleles in the second SNP locus. Denote by μ_*ij*_, *i,j* = (0, 1), the mortality risks for each of four haplotypes. We assume for simplicity that these risks are age-independent. Adding age dependence will not qualitatively change our results. Let *m*_00_(*t*), *m*_10_(*t*), *m*_01_(*t*), and *m*_11_(*t*) be the frequencies of corresponding haplotypes at age *t* ≥ *t*_0_, and μ_00_, μ_10_, μ_01_, μ_11_ be the mortality risks for these haplotypes. The minor allele frequencies *m*_1_(*t*) and *m*_2_(*t*) at any age *t* ≥ *t*_0_ are defined as *m*_1_(*t*) = *m*_10_(*t*) + *m*_11_(*t*) and *m*_2_(*t*) = *m*_01_(*t*) + *m*_11_(*t*). We assume that in the absence of LD at age *t*_0_ the minor allele in the first locus has a positive association with lifespan (i.e., it is a “longevity” allele), and the minor allele in the second locus has a negative association with lifespan (i.e., it is a “vulnerability” allele).

To make this possible we assume that mortality risks for the haplotypes satisfy the inequalities:

(1) $$\begin{array}{l} \mu_{10}<\mu_{00}<\mu_{11}<\mu_{01} \end{array}$$

To minimize the effects of the mathematical representation of the connections between the genetic factors and mortality on the results of our simulation experiment we considered two models of mortality risks. For Model 1, we assumed that:

(2) $$\begin{array}{l} \mu_{10}=\mu_{00}\left(1+R_{1}\right),\mu_{01}=\mu_{00}\left(1+R_{2}\right),\;and\;\mu_{11}=\mu_{00}\left(1+R_{1}+R_{2}\right) \end{array}$$

where *R*_1_ and *R*_2_ could be called the increments to the haplotypes' relative risks associated with the presence of minor alleles in the first and second loci, respectively. To guarantee Equation (1) the values of *R*_1_ and *R*_2_ have to satisfy inequalities:

$$\begin{array}{l} -1<R_{1}<0,R_{2}>0,\;and\;R_{1}+R_{2}>0. \end{array}$$

For Model 2, we assumed that:

(3) $$\begin{array}{l} \mu_{10}=\mu_{00}H_{1},\mu_{01}=\mu_{00}H_{2},\;and\;\mu_{11}=\mu_{00}H_{1}H_{2}. \end{array}$$

These relative risks have to satisfy the inequalities:

$$\begin{array}{l} H_{1}<1,H_{2}>1,\;and\;H_{1}H_{2}>1. \end{array}$$

In our simulation experiments, we fix the initial frequencies *m*_1_(*t*_0_) and *m*_2_(*t*_0_), specify values of *R*_1_ and *R*_2_ (or *H*_1_ and *H*_2_), fix the initial levels of *LD*(*t*_0_), and run the models to calculate the age trajectories of mortality rates for the carriers μ_1_(*t*) and non-carriers μ_0_(*t*) of the minor allele in the first locus. The calculation of the age trajectories of mortality rates for the carriers and non-carriers of the minor allele in the first SNP locus, as well as the age trajectories of other variables, are shown in the Supplementary Materials.

## Results

The results of four simulation experiments with Model 1 are shown in Figure 1. The corresponding model parameters are presented in Table 1.

**Table 1:** **Parameter values used in four simulation experiments with Model 1**.

	***m*_1_(*t*_0_)**	***m*_2_(*t*_0_)**	***LD*(*t*_0_)**	***m*_00_(*t*_0_)**	***m*_10_(*t*_0_)**	***m*_01_(*t*_0_)**	***m*_11_(*t*_0_)**	**μ_00_**	***R*_1_**	***R*_2_**
(A)	0.4	0.4	−0.16	0.2	0.4	0.4	0	0.07	−0.2	0.8
(B)	0.4	0.4	0.23	0.59	0.01	0.01	0.39	0.07	−0.2	0.8
(C)	0.4	0.4	0.13	0.49	0.11	0.11	0.29	0.07	−0.2	0.8
(D)	0.28	0.41	0.165	0.59	0	0.13	0.28	0.07	−0.35	0.4

*m_1_(t_0_) is the initial value of the minor allele frequency at the SNP1 locus; m_2_(t_0_) is the initial value of the minor allele frequency at the SNP2 locus; LD(t_0_) is the initial value of linkage disequilibrium between the SNP1 and SNP2 loci; m_00_(t_0_) is the initial value of the haplotype (0, 0) frequency; m_10_(t_0_) is the initial value of the haplotype (1, 0) frequency; m_01_(t_0_) is the initial value of the haplotype (0, 1) frequency; m_11_(t_0_) is the initial value of the haplotype (1, 1) frequency; μ_00_ is the mortality risk for carriers of haplotype (00); R_1_ and R_2_ are the increments to the haplotypes' relative risks associated with the presence of minor alleles in the first and in the second loci, respectively. Mortality risks for haplotypes (0, 0), (1, 0), (0, 1), and (1, 1) are calculated using equation (2)*.

One can see from Figure 1 and Table 1 that the graphs of the mortality rates shown in Figures 1A–C correspond to the same initial values of the minor allele frequencies *m*_1_(*t*_0_) and *m*_2_(*t*_0_) in the two SNP loci in each of three experiments and the same (constant) values of μ_00_, *R*_1_, and *R*_2_. The latter values are used for calculating mortality risks for haplotypes (Equation 2). The differences in the initial LD levels and in haplotype frequencies are responsible for the radical differences in relationships between mortality rates for carriers and non-carriers of the minor allele at the SNP1 locus (shown in Figure 1). Figure 1A shows that the mortality rate for carriers of the minor allele of SNP 1 is lower than that for non-carriers of this allele, suggesting that this is a “longevity” allele. Figure 1B shows that the mortality rate for carriers of the minor allele of SNP 1 is higher than that for non-carriers of this allele, suggesting that this is a “vulnerability” allele. Figure 1C shows that the mortality rates for carriers and non-carriers of the minor allele of SNP 1 intersect: the harmful effect of the allele on mortality risk at the initial age interval changed to a beneficial one later in life. Figure 1D shows that mortality rates for carriers and non-carriers of the minor allele of SNP 1 may intersect in the opposite way: the beneficial effect of the allele on mortality risk at the initial age interval changed to a harmful one later in life. The results of analyses using Model 2 are similar to those of Model 1. They can be found in Figure 2S in the Supplementary Materials.

## Discussion

In this paper we showed that the finding of an association of lifespan with a functional SNP does not exclude the possibility of non-replication due to differences in initial LD patterns among study populations. The detected associations may differ dramatically if the study populations have different LD patterns around the functional SNP locus. This may happen if the LD area includes one or more additional causal loci affecting the same trait. We showed that in this case the difference in the LD value may change the sign of the estimated genetic association with mortality risks to the opposite direction. The possibility of such a phenomenon was highlighted by Lin et al. (2007) who provided examples of genetic variants that show such “flip-flop” effects in genetic association studies. The authors performed comprehensive simulation analyses of “static” cases in which LD levels influence relative risks of corresponding genetic variants. The results of their study provide us with valuable information for proper interpretation of findings from genetic association studies of risks of diseases whose occurrence does not affect mortality risks. Additional analyses motivated by Lin et al. (2007) showed that differences in frequencies of genetic variants may also produce reversals of the effects of genetic variants on disease risks (Zaykin and Shibata, 2008). We also showed that differences in the LD levels and other parameters of population genetic structure can change the signs of the genetic effects on longevity related traits at different age intervals.

Despite their high relevance for many genetic association studies, the “static” cases of LD effects on relative risks described in Lin et al. (2007) and Zaykin and Shibata (2008) may be less informative when studying the risks of chronic conditions (e.g., CVD, cancer) whose occurrences do influence mortality risk. This is because genetic variants affecting such risks are involved in mortality selection processes in which the most vulnerable individuals in a population birth cohort tend to become sick and die first. This process, in turn, generates age-related changes in genotype (allele) frequencies, in values of disease risks for carriers and non-carriers of selected genetic variants, as well as in mortality risks. In particular, non-monotonic age trajectories of the frequencies of selected alleles or genotypes (Yashin et al., 1999, 2000; Atzmon et al., 2006; Bergman et al., 2007) could result from specific LD patterns. The estimated effects of genetic variants on longevity related traits in populations that have different initial LD levels between selected SNP loci or, more generally, different genetic structures may differ dramatically. The age trajectories of LD values between functional SNP loci may also change with increasing age of the population cohorts.

Recently Ukraintseva et al. (2016) investigated possible causes and mechanisms of paradoxical behaviors of genetic risk factors including age-dependence of the effects of genetic variants on disease and mortality risks. Such effects may result from pleiotropic influences of a genetic variant on chronic health disorders and on aging-related phenotypes. They can also be caused by changes in the epistatic effects on mortality risk or in the effects of interactions of genetic factors with environmental conditions with increasing age. Each of these causes may result in non-replication. In this paper we modeled another possible cause for non-replication resulting from the differences in LD levels between pairs of functional SNP loci, each of which is associated with lifespan, in two populations. Taking the possibility of such effects into account is crucial for proper interpretation of the results of genetic analyses of complex traits.

The results presented in this paper will be highly relevant to genetic studies of human aging, health, and longevity related traits only if the assumed variability in LD values at different locations of the human genome is common in populations used for genetic association studies. Numerous studies show that LD patterns vary from one population to the next at different areas of the human genome. The differences in LD of lipid-associated loci in different study populations and the implications of these differences for genetic analyses across multiple populations were investigated in Teo and Sim (2010). Charles et al. (2014) provided an overview of LD measures used in analyses of population genetic structures and discussed their possible use in GWAS of populations with discordant LD patterns. Sawyer et al. (2005) analyzed LD patterns of selected genomic regions in diverse populations and provided evidence of marked differences in haplotype frequencies and in corresponding LD patterns. Liu et al. (2013) emphasized that genetic analyses of admixed populations may result in confounding due to different patterns of genetic structure. Thomson and colleagues found differences in LD patterns between populations of Hutterites and Europeans (Thompson et al., 2010). The results of Koda and colleagues suggest that some differences in the estimates of risk for coronary heart disease can be explained by population differences in haplotype frequencies of the PON1 haplotypes (Koda et al., 2004). Lohmueller et al. (2006) found genetic variation in the LD patterns in the G-protein coupled receptor kinase 4 (GRK4 gene). The product of this gene inhibits the dopamine receptor D1 (DRD1). The LD patterns were found to be different in SNPs related to 81 osteoporosis candidate genes (Kim et al., 2007). Garner and Slatkin found extensive variation in the LD patterns between a disease locus and one or two marker SNP loci—even for closely linked loci (Garner and Slatkin, 2003). They also found that the distribution of LD patterns between common variants is strongly influenced by ancestral population size.

Thus, the differences in the LD patterns among study populations may result in misleading interpretations of the biological mechanisms involved in trait regulation. Differences in LD are likely to be present in study populations sampled in the U.S. Many such populations contain mixtures of subpopulations that migrated to the U.S. from different parts of the world. In cases where the LD patterns in the study populations do not coincide with those of the specific ethnicities in HAP/MAP or other reference datasets, one may expect the results of genetic association studies to be different. Estimation of the LD patterns around specific SNP loci in the targeted study populations can help one to better understand the sources of inconsistency in the GWAS results.

The fact that the second functional SNP may be unobserved (e.g., not included in the list of SNPs for genotyping) in a given study population makes the results of traditional replication analyses unpredictable and may result in a failure to replicate earlier research findings. To reduce the chances of non-replication due to differences in the LD patterns in studies of independent populations, one has to conduct the replication study using populations with similar patterns of LD around specific targeted loci. This mean that different study populations may be needed for replication of the research results concerning the effects of different SNPs. Non-replication due to other (non-LD) reasons may also occur. Dealing with these other sources of non-replication will require different approaches (Ukraintseva et al., 2016).

**Figure 2:**
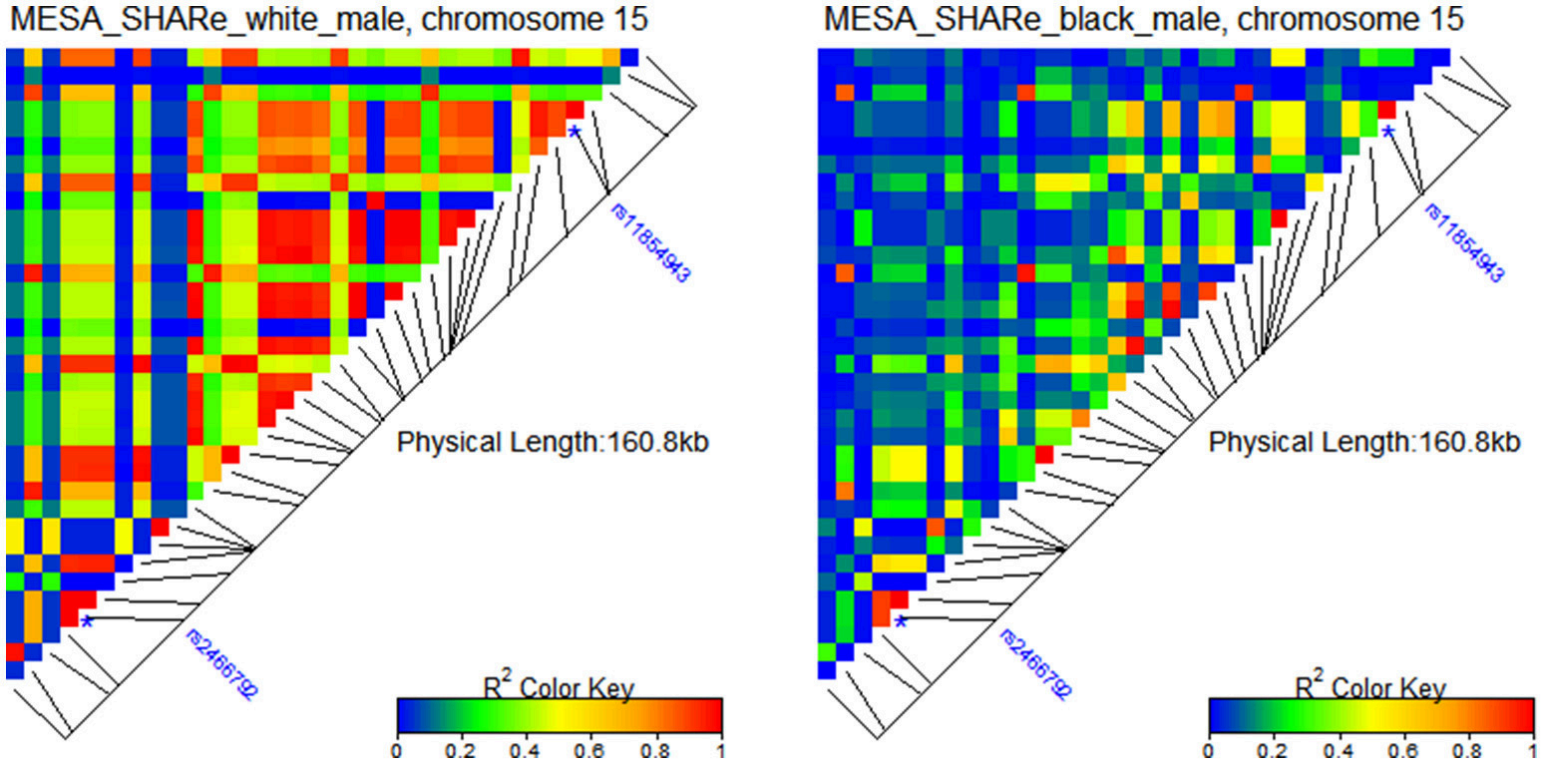
**Patterns of LD around rs2466792 and rs11854943 SNPs in gene FBN1 on chromosome 15 in populations of white and black male participants of MESA**. The SHARe genetic data were used in the LD analyses.

Figure 2 shows two distinct patterns of LD for white and black male participants of the Multi-Ethnic Study of Atherosclerosis (MESA) SNP Health Association Resource (SHARe), available from dbGaP.

One can see from the figure that there are many more SNPs that are in strong LD in the population of white males than in the population of black males from the same study. The two SNPs shown in this figure are rs2466792 and rs11854943 in gene FBN1 on chromosome 15. The minor allele of the rs2466792 SNP (MAF 43.6%) reduces mortality risk (relative risk HR = 0.7; *p*-value 0.03) among black males MESA participants. The minor allele of the rs11854943 SNP (MAF 10.7%) increases mortality risk (relative risk HR = 1.5, *p*-value 0.04) among black males MESA participants. Neither allele shows an association with lifespan among white male MESA participants.

Although, the causes of the different effects of selected SNPs on mortality risks between black and white males may include influences of gene-gene and gene-environment interactions which are not estimated in these analyses, the difference in LD could also make substantial contributions to these differences, as illustrated in Figure 3S. This figure shows age patterns of mortality rates for carriers and non-carriers of minor alleles in populations with different levels of LD. Panels “e, f” of Figure 3S in this figure correspond to populations with higher and lower LD values, respectively. The intersection of age patterns of mortality risks for carriers and non-carriers of the minor allele in panel “e” of Figure 3S indicates that the estimate of relative risk using the Cox regression model over the entire age interval is likely to be close to one (because differences between the curves will tend to compensate each other), i.e., analyses will not show statistically significant associations of this genetic variant with lifespan. The two non-intersecting age patterns of mortality risks for carriers (red line) and non-carriers (blue dashed line) of the minor allele in the population with the lower initial value of LD (panel “f” of Figure 3S) indicate that the differences in hazard rates and hence in survival functions between these groups can be estimated by statistical methods (e.g., using a Cox-type regression model).

It is important to note that differences in the LD patterns may be responsible for non-replication of the results of (non-genetic) epidemiological studies. Such confounding may take place when two or more causal SNP loci influence different phenotypes and are in LD (Aissani, 2014). In this case, one phenotype will always show its association with the other one in epidemiological studies although such a connection may be not causal but induced instead by the LD between the corresponding SNP loci. Further, the use of one such phenotype as a covariate in a GWAS of the second phenotype may substantially modify the estimate of genetic association with the second trait. Different patterns of LD among study populations are expected to produce different confounding effects, jeopardizing proper interpretation of the results of epidemiological studies.

The effect of the minor allele of a causal SNP (SNP1) on lifespan is considered to be “pure” when it is estimated in a population whose genetic structure does not have LD between SNP1 and any other causal SNP. In the absence of other (e.g., non-genetic) confounders this effect characterizes the biological connections between variations in SNP1 and lifespan. If in some other population the causal SNP (SNP1) is in strong LD with another causal SNP (SNP2), then its estimated effect on lifespan in a new genetic association study is confounded and may be radically different from the pure effect (e.g., have the opposite sign). This confounded effect of SNP1 on lifespan may be successfully replicated in the new genetic association study if the level of LD between SNP1 and SNP2 and other parameters of genetic structure in both populations are about the same. Such replication, however, does not guarantee proper interpretation of the effect of SNP1 on lifespan because it is confounded by the effect of SNP2 and depends on the LD level between two SNPs. This also means that analyses of next (third) population with a different LD level between SNP1 and SNP2 may not replicate the results of the two earlier studies. One, however, cannot say that the results of the first two analyses are “more correct” than the findings from the third study because both are confounded by the LD with another causal SNP. The estimates of the pure effect of SNP1 on lifespan can be obtained from the data on the initial population in which SNP1 and SNP2 were not in LD.

Better understanding of how human genes regulate relationships among aging, health, and longevity related traits will contribute to development of intervention strategies aiming to increase healthy lifespan and reduce the burden of chronic non-communicable diseases. A number of promising genetic associations with human longevity related traits were detected in GWAS and confirmed in other populations (Willcox et al., 2008; Deelen et al., 2011; Nebel et al., 2011; Flachsbart et al., 2013; Soerensen et al., 2013, 2015; Broer et al., 2015). At the same time, numerous attempts to replicate many other detected associations with these traits failed (Novelli et al., 2008). Elbaz and colleagues could not replicate 13 SNPs significantly associated with Parkinson's disease (Elbaz et al., 2006). Campa and colleagues could not replicate seven loci associated with the risk of pancreatic cancer detected in studies of two Asian populations (Campa et al., 2013). Suarez–Gestal and colleagues were not able to replicate significant associations between 16 SNPs and responses to specific treatments of rheumatoid arthritis (Suarez-Gestal et al., 2010). Nemr and colleagues could not replicate association of EXT2 genetic variants with the risk of type 2 diabetes in a population of Lebanese Arabs (Nemr et al., 2013). Such persistent non-replication of the results of genetic association studies raises legitimate questions about the factors and mechanisms responsible. Previous research has identified a number of conditions that may contribute to the lack of replication (Ioannidis, 2007; Kavvoura and Ioannidis, 2008; Kraft et al., 2009; Ukraintseva et al., 2016). In this paper, we demonstrated that differences in genetic structures between various study populations can make substantial contributions to the non-replication of the results of genetic association studies of human longevity.

## Author contributions

AY and SU conceived the paper and wrote the first draft. IZ developed software for simulations and together with AY performed simulation experiments. LA and KA performed LD analyses. ArY, KA, and ES provide valuable comments and suggestions and together with DW, MK, AK, IK, IA make valuable contributions to the discussion section. All coauthors participated equally in preparing the ultimate version of the manuscript and Supplementary materials.

### Conflict of interest statement

The authors declare that the research was conducted in the absence of any commercial or financial relationships that could be construed as a potential conflict of interest.
