# Supplementary material for: Pure and Confounded Effects of Causal SNPs on Longevity: Insights for Proper Interpretation of Research Findings in GWAS of Populations with Different Genetic Structures
Source: Front Genet. 2016 Nov 8;7:188. doi: 10.3389/fgene.2016.00188 (PMC5099244; doi:10.3389/fgene.2016.00188)
Supplement: Supplementary file 1 [file DataSheet1.pdf]

\

Supplementary Data

Supplementary Figure 1S.

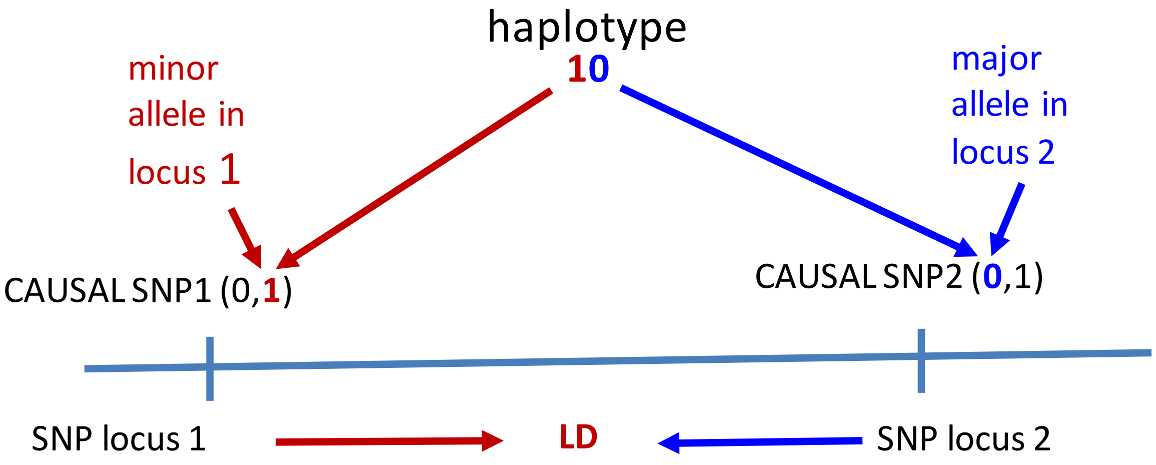

Figure 1S. Diagram illustrating connection between two causal SNP loci. Notation "0" corresponds to the major allele in each locus. Notation "1" is used for the minor allele in each locus. The two loci have four haplotypes: (0,0), (1,0), (0,1), and (1.1). We are interested in mortality rates for carriers and non-carriers of minor allele in locus 1.

Supplementary Figure 2S.

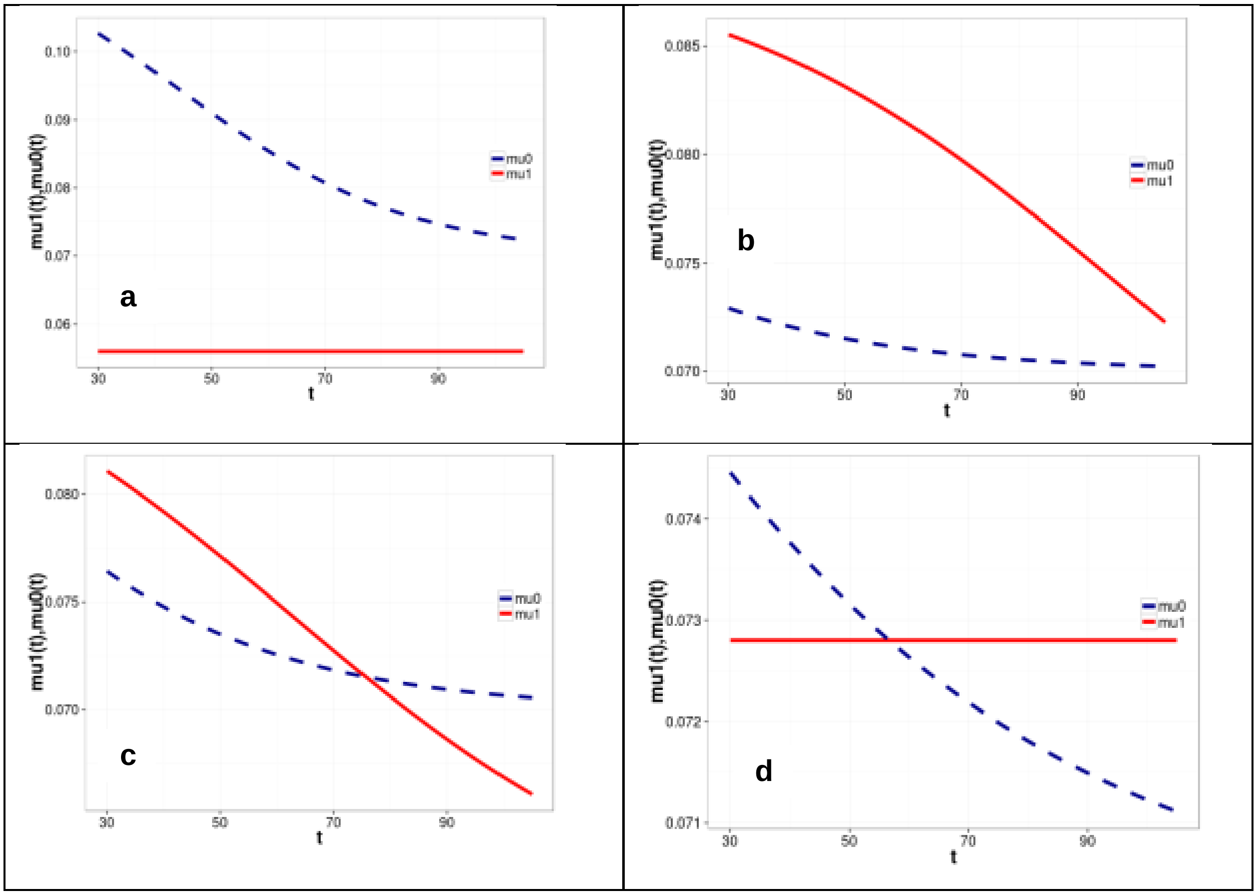

Figure 2S. Graphs of mortality rates for carriers (solid line) and non-carriers (dashed line) of the minor allele at the first SNP locus corresponding to different levels of LD between the two loci in simulation experiments with Model 2. Model parameters corresponding graphs shown in panels **a, b, c,** and **d** are represented in Table 1S in rows (SA), (SB), (SC) and (SD) respectively.

Supplementary Table 1S. Parameters used in four simulation experiments with Model 2. $m_1(t_0)$ is the initial value of minor allele frequency in SNP1 locus; $m_2(t_0)$ is the initial value of minor allele frequency in SNP2 locus; $LD(t_0)$ is the initial value of linkage disequilibrium between SNP1 and SNP2 loci; $m_{00}(t_0)$ is the initial value of haplotype (0,0) frequency; $m_{10}(t_0)$ is the initial value of haplotype (1,0) frequency; $m_{01}(t_0)$ is the initial value of haplotype (0,1) frequency; $m_{11}(t_0)$ is the initial value of haplotype (1,1) frequency; μ_{00} is the mortality risk for carriers of haplotype (0,0); H_1 and H_2 are relative risks of haplotypes (1,0) and (0,1)

	$m_1(t_0)$	$m_2(t_0)$	$LD(t_0)$	$m_{00}(t_0)$	$m_{10}(t_0)$	$m_{01}(t_0)$	$m_{11}(t_0)$	μ_{00}	H_1	H_2
(SA)	0.4	0.4	-0.16	0.2	0.4	0.4	0	.07	0.85	1.5
(SB)	0.4	0.4	0.19	0.55	0.05	0.05	0.35	.07	0.85	1.5
(SC)	0.4	0.4	0.13	0.49	0.11	0.11	0.29	.07	0.85	1.5
(SD)	0.34	0.48	0.177	0.52	0	0.14	0.34	.07	0.8	1.3

Supplementary Note 1. In the second model we assume that $\mu_{10} = \mu_{00}H_1$, $\mu_{01} = \mu_{00}H_2$, and $\mu_{11} = \mu_{00}H_1H_2$, where H_1 and H_2 are relative risks of haplotypes (1,0) and (0,1) with respect to haplotype (0,0). Let as assume that $H_1 < 1$, and $H_2 > 1$. In this case $\mu_{10} < \mu_{00} < \mu_{11} < \mu_{01}$. One can see from **Figure 2S** and **Table 1S** that the graphs of the mortality rates shown in panels "**a**", "**b**", and "**c**" correspond to the same values of the initial allele frequencies in each of the three experiments and the same mortality risks for haplotypes. The differences in the initial LD levels are responsible for the radical differences in the relationships between mortality rates for carriers and non-carriers of the minor allele at SNP 1 locus. Panel "**a**" shows that the mortality rate for carriers of the minor allele of SNP 1 is lower than that for non-carriers of this allele, which suggest that this is a "longevity" allele. Panel "**b**" shows that mortality rate for carriers of the minor allele of SNP 1 is higher than that for non-carriers of this allele, which suggest that this is a "vulnerability" allele. Panel "**c**" shows that mortality rates for carriers and non-carriers of the minor allele of SNP 1 intersect: the harmful effect of the allele on mortality risk at the initial age interval changed to a beneficial one later in life. Panel "**d**" shows that the mortality rates for carriers and non-carriers of the minor allele of SNP 1 may intersect in the opposite way: the beneficial effect of the allele on mortality risk at the initial age interval changed to a harmful one later in life.

Supplementary Figure 3S.

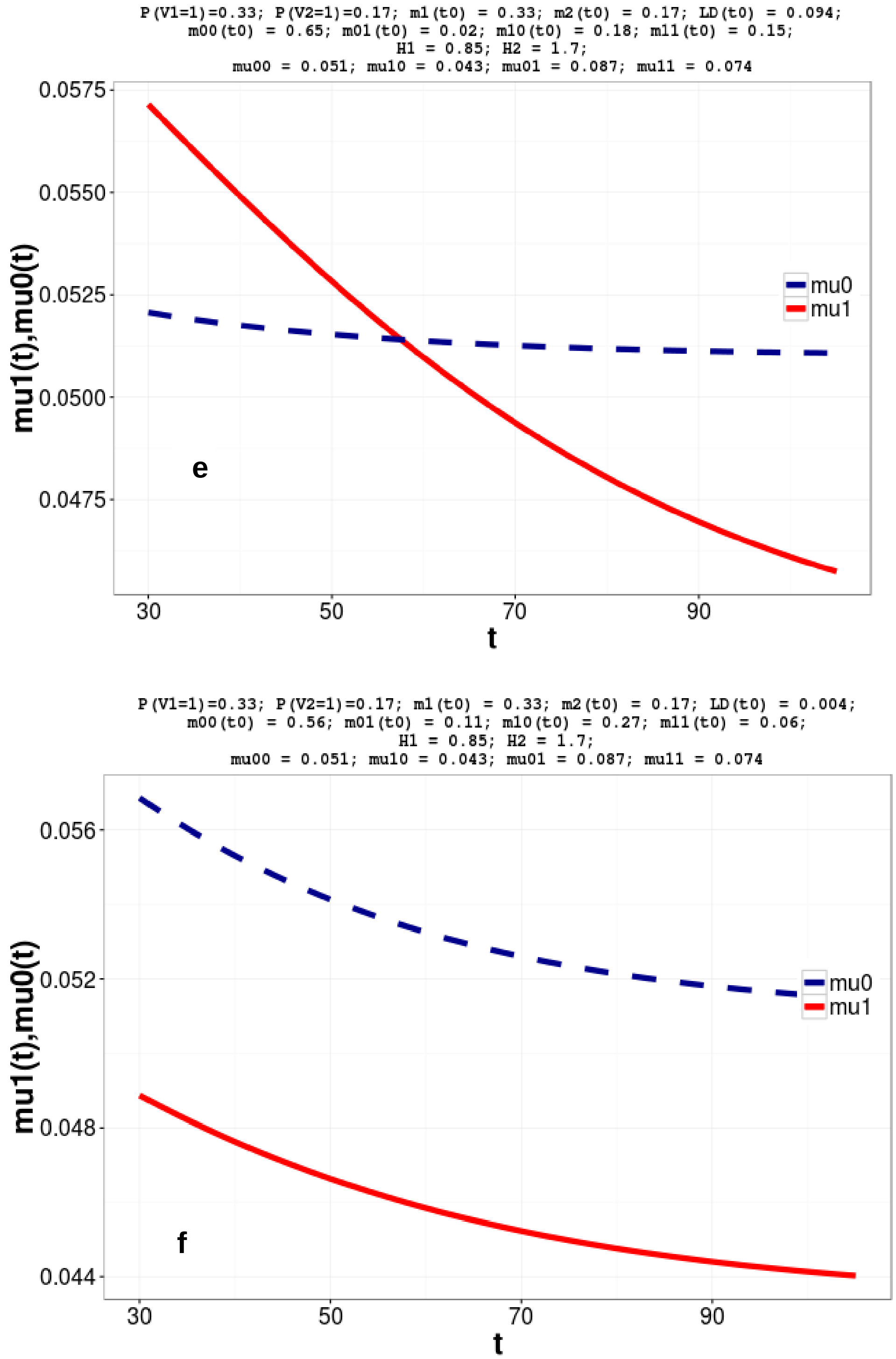

Figure 3S. The results of simulation experiments with Model 2 that show how difference in LD levels between two detected SNPs in black and white males (see Figure 2) may produce differences in estimated effects of SNP1 on lifespan (mortality risk). The parameters of Model 2 used in these experiments are described in **Table 2S.**

Panels **"e"** and **"f"** correspond to the values of $LD(t_0)$ =0.094 and $LD(t_0)$ =0.004, respectively. The intersection of age trajectories of mortality risks for carriers (red line) and non-carriers (blue dashed line) of minor allele (panel **"e"**) indicates that the estimate of relative risk using say the Cox's regression model at the entire age interval is likely to be close to one, i.e., analyses will not show statistically significant association of this genetic variant with lifespan in population with $LD(t_0)$ =0.094. The two non-intersecting age patterns of mortality risks for carriers (red line) and non-carriers (blue dashed line) of minor allele in population with $LD(t_0)$ =0.004 (panel **"f"**) indicate that the use of Cox-type regression model will be able to show that relative mortality risk of carriers of minor allele is lower than that of non-carriers.

Supplementary Table 2S. The values of model parameters used in two simulation experiments with Model 2 whose results are shown in Figure 3S. Rows SE and SF correspond to panels **"e"** and **"f"**, respectively. Notations for model parameters are the same as in Table 1S.

	$m_1(t_0)$	$m_2(t_0)$	$LD(t_0)$	$m_{00}(t_0)$	$m_{10}(t_0)$	$m_{01}(t_0)$	$m_{11}(t_0)$	μ_{00}	H_1	H_2
(SE)	0.33	0.17	0.094	0.65	0.18	0.02	0.15	0.051	0.85	1.7
(SF)	0.33	0.17	0.004	0.56	0.27	0.11	0.06	0.051	0.85	1.7

One can see from **Table 2S** that the two experiments differ in the initial values of LD levels. These differences, in turn, are generated by differences in the initial values of haplotype frequencies. The initial frequencies of minor alleles in the two loci $m_1(t_0)$ and $m_2(t_0)$, as well as mortality risks for each haplotype are the same.

Supplementary Note 2: Haplotype and allele frequencies. Denote by $m_{ij}(t), i, j = 0{,}1$ the frequencies of haplotypes (1,1), (1,0), (0,1), and (0,0) in a population cohort at age t.

$$m_{11}(t) = P(V_1 = 1, V_2 = 1 | T > t),$$

$$m_{10}(t) = P(V_1 = 1, V_2 = 0 | T > t),$$

$$m_{01}(t) = P(V_1 = 0, V_2 = 1 | T > t),$$

$$m_{00}(t) = P(V_1 = 0, V_2 = 0 | T > t).$$

The frequencies of a minor allele at the first and at the second loci are

$$m_1(t) = m_{10}(t) + m_{11}(t) = P(V_1 = 1 | T > t)$$

and

$$m_2(t) = m_{01}(t) + m_{11}(t) = P(V_2 = 1 | T > t),$$

respectively. The measure of LD is defined as

$$LD(t) = m_{11}(t) - m_1(t)m_2(t)\,.$$

If haplotypes (1,1), (1,0), (0,1), and (0,0) influence mortality risk, the corresponding haplotype and allele frequencies as well as the LD measure will change with increasing age. These changes are also related to the age trajectories of survival functions for carriers and non-carriers of the corresponding haplotypes or minor alleles. To describe these changes we need to specify mortality risks for carriers of each haplotype and initial values of the respective frequencies.

Supplementary Note 3: Mortality risks and survival functions for carriers of different haplotypes. Let $\mu_{10}, \mu_{00}, \mu_{11}$ $\mu_{10} < \mu_{00} < \mu_{11} < \mu_{01}$ and μ_{01} be mortality risks for carriers of the corresponding haplotypes. We assume that in the absence of LD the minor allele in locus 1 is a "longevity" allele, and the minor allele at locus 2 is a "vulnerability" allele. We will consider two mortality models for carriers of different haplotypes. In the first $\mu_{10} = \mu_{00}(1+R_1)$, $\mu_{01} = \mu_{00}(1+R_2)$, and $\mu_{11} = \mu_{00}(1+R_1+R_2)$, where R_1 and R_2 are increments of haplotypes' relative risks associated with the presence of minor alleles in the first and in the second loci, respectively. We assume that R_1 and R_2 satisfy inequalities $-1 < R_1 < 0$, $R_2 > 0$, and $R_1 + R_2 > 0$. In the second model we assume that $\mu_{10} = \mu_{00}H_1$, $\mu_{01} = \mu_{00}H_2$, and $\mu_{11} = \mu_{00}H_1H_2$, where H_1 are H_2 relative risks of haplotypes (1,0) and (0,1) with respect to haplotype (0,0). Let as assume that $H_1 < 1$, and $H_2 > 1$. In this case $\mu_{10} < \mu_{00} < \mu_{11} < \mu_{01}$. So in the absence of LD between loci the frequency of the minor allele in the first loci will monotonically increase and mortality rate for carriers on minor allele in the first locus will be smaller than that for non-carriers of this allele. The survival functions for carriers of the corresponding haplotypes are

$$S_{01}(t) = P(T > t \mid V_1 = 0, V_2 = 1, T > t_0) = e^{-\mu_{01}(t-t_0)}$$

$$S_{10}(t) = P(T > t \mid V_1 = 1, V_2 = 0, T > t_0) = e^{-\mu_{10}(t-t_0)}$$

$$S_{11}(t) = P(T > t \mid V_1 = 1, V_2 = 1, T > t_0) = e^{-\mu_{11}(t-t_0)}$$

$$S_{00}(t) = P(T > t \mid V_1 = 0, V_2 = 0, T > t_0) = e^{-\mu_{00}(t-t_0)}\,.$$

Here t_0 is the starting age of observation in the cohort.

Supplementary Note 4: Dynamics of genetic frequencies and survival functions. Let $m_{ij}(t_0), i, j = 0,1$ be the initial values of the genetic frequencies for haplotypes (1,1), (1,0), (0,1), and (0,0) . Then

$$m_{00}(t) = P(V_1 = 0, V_2 = 0 \mid T > t) = \frac{m_{00}(t_0)e^{-\mu_{00}(t-t_0)}}{\sum_{i=0}^{1}\sum_{j=0}^{1} m_{ij}(t_0)e^{-\mu_{ij}(t-t_0)}}$$

$$m_{10}(t) = P(V_1 = 1, V_2 = 0 | T > t) = \frac{m_{10}(t_0)e^{-\mu_{10}(t-t_0)}}{\sum_{i=0}^{1}\sum_{j=0}^{1} m_{ij}(t_0)e^{-\mu_{ij}(t-t_0)}}$$

$$m_{01}(t) = P(V_1 = 0, V_2 = 1 | T > t) = \frac{m_{01}(t_0)e^{-\mu_{01}(t-t_0)}}{\sum_{i=0}^{1}\sum_{j=0}^{1} m_{ij}(t_0)e^{-\mu_{ij}(t-t_0)}}$$

$$m_{11}(t) = P(V_1 = 1, V_2 = 1 | T > t) = \frac{m_{11}(t_0)e^{-\mu_{11}(t-t_0)}}{\sum_{i=0}^{1}\sum_{j=0}^{1} m_{ij}(t_0)e^{-\mu_{ij}(t-t_0)}}.$$

For the frequencies of the minor alleles in the first and in the second loci we have

$$m_1(t) = P(V_1 = 1 | T > t) = \frac{m_{10}(t_0)e^{-\mu_{10}(t-t_0)} + m_{11}(t_0)e^{-\mu_{11}(t-t_0)}}{\sum_{i=0}^{1}\sum_{j=0}^{1} m_{ij}(t_0)e^{-\mu_{ij}(t-t_0)}}$$

$$m_2(t) = P(V_2 = 1 | T > t) = \frac{m_{01}(t_0)e^{-\mu_{01}(t-t_0)} + m_{11}(t_0)e^{-\mu_{11}(t-t_0)}}{\sum_{i=0}^{1}\sum_{j=0}^{1} m_{ij}(t_0)e^{-\mu_{ij}(t-t_0)}}.$$

The survival function for carriers and non-carriers of the minor allele in the first locus are

$$S_{1carr}(t) = \frac{m_{10}(t_0)}{m_{10}(t_0) + m_{11}(t_0)} e^{-\mu_{10}(t-t_0)} + \frac{m_{11}(t_0)}{m_{10}(t_0) + m_{11}(t_0)} e^{-\mu_{11}(t-t_0)}$$

$$S_{1non}(t) = \frac{m_{01}(t_0)}{m_{01}(t_0) + m_{00}(t_0)} e^{-\mu_{01}(t-t_0)} + \frac{m_{00}(t_0)}{m_{01}(t_0) + m_{00}(t_0)} e^{-\mu_{00}(t-t_0)}.$$

The mortality rates for carriers and non-carriers of minor allele in the first SNP locus can be calculated as follows:

$$\mu_1(t) = \mu_{10}\frac{m_{10}(t)}{m_{10}(t) + m_{11}(t)} + \mu_{11}\frac{m_{11}(t)}{m_{10}(t) + m_{11}(t)}$$

$$\mu_0(t) = \mu_{01}\frac{m_{01}(t)}{m_{01}(t) + m_{00}(t)} + \mu_{00}\frac{m_{00}(t)}{m_{01}(t) + m_{00}(t)}$$
